# Do congenitally blind people have visual dreams?

**DOI:** 10.5935/1984-0063.20200068

**Published:** 2021

**Authors:** Michael J. O. Andrade

**Affiliations:** Universidade do Estado de Minas Gerais, Psicologia - Divinópolis - MG - Brazil.

**Keywords:** Sleep, Dreams, Deaf-Blind Disorders

## Abstract

The predominance of visual content in dreams raises a fundamental issue in the formation of images and for the construction of ideas based on the activity of the visual cortex in people with visual impairments. The central question for students of the visual system and for dream connoisseurs is: to what extent the absence or loss of vision will affect the sensory sensitivity for dream construction. Sensory modalities other than vision (tactile and auditory) can inﬂuence the functional development of the occipitotemporal visual system in the absence of visual stimulation early in life. What I mean is that blind individuals have signiﬁcantly less visual capacity, but they also have an increase in the number of auditory, tactile, olfactory, and gustatory sound impressions.

The predominance of visual content in dreams raises a fundamental question about the ability to form images and build visual memories in the activity of the visual cortex in healthy people. However, several questions can be posed on the material of dreams of people with innate or acquired blindness, that is, congenitally blind people or those who acquire blindness due to a socio-environmental accident. Furthermore, how can visual images be constructed in the dreams of people who have never had visual experiences? The central question of this study is to know to what extent the total or partial absence or sensory loss of vision causes cognitive effects in the dream- building process.

Following the reasoning that the circadian rhythm is controlled by the biological clock, this, in turn, is synchronized by light over a period of 24 hours. This is so because light stimulates specific cells located on the retina that send neural projections directly to the suprachiasmatic nucleus (structure located in the hypothalamus). From the suprachiasmatic nucleus, projections depart from several regions that control physiological, psychological, and cognitive variables. Thus, the sleep-wake cycle in humans is expressed by sleep during the night and wake during the period. Although the role of the absence of light perception and what are the mechanisms of the free- flowing pattern of circadian rhythm involved in the processing of the sleep cycle and wakefulness of blind people are not entirely understood, it is known that there is a higher prevalence of sleep disturbance, difficulty in sleeping, initiate sleep, stay awake during sleep, and reported waking up very early^1^. Recent studies describe the relationship between visual activity and eye movements associated with electroencephalographic features during the sleep of blind people, and these authors refute the possibility of every the building of dreams in blind people^2^.

Important points about the sensorial construction of dreams and the frequency of nightmares were seen in congenital blind and late blind individuals^2^. The authors conducted the continuity hypothesis by Hall and Van de Castle who suggested that the content of dreams is a continuum between the awakening of cognition and behavior, that is, visual deprivation causes a reorganization of the sensory composition related to the emotional and thematic contents of dreams. Thus, we started to ask questions about the possible prevalence of non-visual sensory impressions in the dreams of the blind compared to that of the non-blind.

Initially, the authors take into account that individuals who are blind after 7 years of age retain visual images in their dreams, although how they acquire the sleep behavior learning process remains unclear. On the other hand, the general level of emotions in the dreams of blind individuals is similar to or lower than that of non-blind individuals. In general, this study suggested that none of the participants with total blindness or without residual luminous perception had a visual component in their dreams. However, participants with partial blindness who retain residual colors and perception of light were able to form some visual impressions in dreams.

The sleep architecture and microstructure of congenital and acquired blind people, even at an early age, show compensatory cortical plasticity, not only in the visual cortical areas but also throughout the brain throughout brain development and maturation^3^. It is possible to verify several electro-encephalographic microstructural components of sleep, both during rapid eye movement (REM) sleep and non-REM sleep. During wakefulness, alpha occipital oscillations were minor or absent in blind individuals, this type of wave is an important graph for visual attentional processes. Even when awake, alpha occipital activity is lower in blind individuals, both in early and late adolescence. Differences in the spectral power of non-REM sleep between early and late blinds may reflect the gain or loss of altered cortical network activities in blindness.

Another important point related to the electroencephalographic trace of blind subjects is the role of eye movements in the construction of dreams^4^. Rapid eye movements were absent during the dream periods of congenital and late blind men. These authors have suggested that the non-use of the nervous pathways involved in the execution of blind eye movements causes the absence of eye movements in blind subjects to lose the ability to construct visual images.

Recently, objective evidence has been provided that individuals who have never had visual experiences can dream of virtual images, which are probably mediated by the activation of the cortical areas responsible for visual representations^5^. This cross-modal effect is related to the facilitation of auditory and tactile inputs to process information in the brains of congenitally blind individuals for the formation of dreams^6^.

Certainly, elements of the seer’s cognition come into the composition of the mental images of precocious blind people. Thus, it is believed that visual elements tend to be composed with tactile elements and other senses. The brain activity associated with each separate sensory component can contribute to the general consolidation of episodic memory, and some of these processes may be involved in dreams. A fundamental point is that the activation of the occipitotemporal cortex in congenitally blind individuals can be shaped by other sensory abilities, particularly by touch. Sensory modalities other than vision, tactile, and auditory, for example, can influence the functional development of the occipitotemporal visual system in the absence of visual stimulation early in life. Thus, it is concluded that blind individuals have significantly less visual capacity, but also have an increase in the number of auditory, tactile, olfactory, and gustatory sound impressions.

Dreams have multiple layers of meaning that are not only determined by real, external conditions, but also often relate to internal life and past experiences in complex ways (please note that information flows from the cortex to the hippocampus during REM). In this regard, the identification of particular sensory perceptions can be considered a predetermined property of specific cortical areas. In the congenitally blind, the interactions between non-visual systems and predetermined cortical areas to mediate visual perception are established by the cross-modal expansion of non-visual inputs.

It is important that further studies clarify the electroencephalographic cortical activity during sleep and dreams phase of blind individuals. This perspective may contribute to understanding the developmental neural processing and the possible cognitive skills associated with the formation of images and the use of memories blind people.
